# Supplementary material for: Integrating bioinformatics and machine learning to elucidate the role of protein glycosylation-related genes in the pathogenesis of diabetic kidney disease
Source: PLoS One. 2025 Aug 18;20(8):e0329640. doi: 10.1371/journal.pone.0329640 (PMC12360563; doi:10.1371/journal.pone.0329640)
Supplement: Attachment — Submitted filename: Response to Reviewers.docx [file pone.0329640.s003.docx]

Dear Editors and Reviewers,

We sincerely appreciate the reviewers' constructive comments and the editor's thoughtful suggestions. We have carefully revised the manuscript based on these feedbacks. Below is our point-by-point response to the reviewers' comments.

Reviewer #1

1. Data Availability and Transparency

The raw gene expression datasets (GSE96804, GSE104948-GPL22945, GSE30528) analyzed in this study are publicly available through the NCBI Gene Expression Omnibus (GEO) database. Glycosylation-related gene sets were retrieved from the MSigDB database. To ensure reproducibility, the batch-corrected and normalized merged dataset (merge_DN.txt) has been uploaded as supplementary material. This approach aligns with similar studies that utilize standardized bioinformatics workflows for data integration and normalization.

For experimental validation, raw qPCR Ct values, fold-change calculations, and histopathology data from the DKD mouse model are available upon reasonable request. raw qPCR Ct values has been uploaded as supplement. A formal Data Availability Statement (DAS) will be included in the final manuscript, directing readers to supplementary files for access to processed datasets and experimental data.

2. Statistical Rigor in Differential Gene Expression Analysis

Pre-correction PCA: PC1 explained 91.6% of the variance, indicating strong technical batch effects. PC2 accounted for 6.6% of the variance. Post-correction PCA: After applying the limma and sva packages, PC1 variance dropped to 13.8%, and PC2 increased to 9.1%, reflecting reduced technical variability and improved alignment of samples based on biological differences. These results confirm that batch correction effectively mitigated non-biological variability, allowing downstream analyses to focus on biologically relevant signals. We will revise the Methods section to include these quantitative details and explained pre- and post-correction.

We acknowledge that this threshold balances sensitivity (detecting true positives) and specificity (minimizing false positives) and have strengthened our rationale as follows: 1. Biological Relevance: In complex diseases like DKD where subtle changes may lead clinical significance. Similar thresholds(|logFC| > 1 and |logFC| > 0.5) have been successfully applied in prior studies of DKD, supporting our methodological consistency^[1][2]^. 2. Statistical Rigor: We combined this threshold with an adjusted p-value < 0.05 (Benjamini-Hochberg correction) to ensure robust statistical significance while controlling for false discoveries. 3. Downstream Validation: The selected DEGs demonstrated strong enrichment in DKD-relevant pathways (e.g., PI3K-Akt signaling, ECM remodeling) and were validated via qPCR and ROC analysis (AUC > 0.7 for hub genes). This confirms the functional relevance of the threshold.

3. Enrichment Analysis (GO & KEGG Pathways)

In all GO and KEGG analyses, we explicitly applied Benjamini-Hochberg (BH) correction to control the false discovery rate (FDR), as stated in the revised Methods (Section 1.2). Adjusted p-values (p.adjust < 0.05) were used to define statistical significance for both DEGs and gly-DEGs. For example, gly-DEGs were filtered with p.adjust < 0.05 (Figures 3D, 4C), and pathway enrichments (e.g., heparan sulfate biosynthesis, p.adjust = 0.003) adhere to this threshold. This ensures rigorous control of multiple hypothesis testing.

To complement overrepresentation analysis (ORA), GSEA was performed on hub genes using Reactome pathways (Section 1.9). Key pathways (e.g., extracellular matrix remodeling, NES = 2.15, p.adjust = 0.008) validated ORA results, demonstrating consistency between methods. Additionally, single-gene GO/KEGG analyses (Supplementary File S2) further confirmed hub genes’ roles in immune infiltration and fibrosis (e.g., leukocyte migration, p.adjust = 0.003). These integrated approaches strengthen the reliability of our findings.

4. Machine Learning-Based Hub Gene Selection

Our study prioritized glycosylation-related molecular mechanisms in DKD, with immune infiltration serving as a secondary exploratory component. Given the non-tumor context and limited sample size (n=108 total samples across datasets), we employed ssGSEA—a widely used and robust method for immune cell scoring—to avoid overcomplicating the analysis. While tools like CIBERSORT or xCell are valuable, they require larger sample sizes for stable deconvolution and are optimized for tumor microenvironments. Our preliminary results (Supplementary Figure S3) highlighted significant differences in macrophage/neutrophil activity between DKD and controls, validated by immunohistochemistry (Section 2.8). These findings align with the study’s focus on glycosylation-driven pathways rather than immune heterogeneity.

The reviewer’s concern about multiple testing correction is valid. However, in our immune correlation analysis (Section 1.9), strict FDR adjustment (e.g., Benjamini-Hochberg) was intentionally omitted due to the small sample size and exploratory nature of this analysis. Applying FDR correction here would have increased the risk of false negatives, masking biologically plausible immune-gene correlations (e.g., hub genes like S100A12 and EXT1 showed trends with neutrophil infiltration). We acknowledge this limitation in the revised Discussion (Section 4.4) and emphasize that future studies with larger cohorts will incorporate cross-method validation (e.g., TIMER) and stringent FDR control to enhance robustness.

5. Immune Cell Infiltration Analysis (ssGSEA)

Our study focused on glycosylation-related mechanisms in DKD pathogenesis, with immune infiltration as an exploratory component. Given the non-tumor context and limited sample size (n=108 across datasets), we selected ssGSEA for immune scoring due to its robustness in small cohorts and avoidance of tumor-specific deconvolution biases inherent to tools like CIBERSORT/xCell. This approach prioritized simplicity and relevance to our biological focus (Section 1.9).

The correlation analysis between hub genes and immune cells (Section 2.9) intentionally omitted strict FDR correction. In small cohorts, FDR adjustment can overly suppress biologically plausible signals (e.g., S100A12-neutrophil correlations, p=0.02 uncorrected). We have explicitly acknowledged this limitation in the revised Discussion (Section 4.4) and emphasized that future studies with larger cohorts will apply cross-method validation (e.g., TIMER) and stringent FDR control.

While alternative methods (e.g., CIBERSORT) were not compared, our ssGSEA results aligned with immunohistochemical validation of immune infiltration (Figure 8, CD45+ cell quantification). This consistency supports the validity of our exploratory findings within the study’s scope.

6. Experimental Validation (qPCR & Mouse Model)

We used C57BKS db/db mice, a widely recognized model for DKD, and confirmed the successful establishment of the DKD model through immunohistochemistry. Compared to the high genetic heterogeneity in DKD patients, this model has lower genetic variability, and three samples per group meet the basic statistical requirements.

The qPCR results are consistent with the bioinformatics predictions, supporting the statistical significance among the groups with the current sample size. This indicates that our findings are reliable and representative.

We acknowledge that a larger sample size would enhance statistical power. However, due to the 16-week duration of DKD model development and ethical/resource constraints, expanding the cohort is challenging. We commit to supplementing the qPCR results with additional samples after completing the ongoing 16-week DKD model replication. Additionally, we will consider making the raw qPCR data available.

Reviewer #2

1. Clarity and Terminologies

Comment 1.1: Title adjustment to reflect focus on glycosylation-related genes

Response: We agree with the reviewer’s suggestion. The title has been revised to better reflect the gene-centric focus of the study: Revised Title: "Integrating Bioinformatics and Machine Learning to Elucidate the Role of Glycosylation-Related Genes in the Pathogenesis of Diabetic Kidney Disease"

Comment 1.2: Clarify "pathogenesis" vs. "progression"

Response: We acknowledge the distinction between "pathogenesis" (mechanisms underlying disease onset) and "progression" (disease advancement). Our study primarily investigates molecular mechanisms driving DKD development, not longitudinal changes across stages. We have revised the terminology throughout the manuscript:

Abstract: "This study aimed to investigate the role of aberrant protein glycosylation in DKD pathogenesis and its association with gene expression changes..."

Introduction: "...exploring the role of dysregulated glycosylation in DKD pathogenesis..."

Comment 1.3: Use of "abnormal protein glycosylation"

Response: We thank the reviewer for highlighting this critical point. We have clarified that the study focuses on aberrant glycosylation (a pathological process) rather than physiological glycosylation. Revisions include:

Abstract (Conclusions): "This study highlights abnormal protein glycosylation as a key player in DKD..."

Comment 1.4: Simplify redundant phrases

Response: We removed redundant terms like "modification" where appropriate to enhance clarity:

Original: "protein glycosylation modification"

Revised: "protein glycosylation"

2. Methods

Comment 2.1: Training/testing splits and cross-validation

Response: Due to the limited sample size of publicly available DKD datasets (total n=108n=108), splitting the data into training and testing sets would have reduced statistical power. Instead, we employed 10-fold cross-validation during model training to ensure robustness. This is now explicitly stated in the Methods section (Section 1.5): "LASSO, SVM, and random forest models were trained using 10-fold cross-validation to minimize overfitting and enhance generalizability."

The limitation of sample size is acknowledged in the Discussion: "Future studies should validate these hub genes in larger cohorts to address the current sample size constraints."

3. Results

Comment 3.1: Separate observations from implications

Response: We restructured the Results section to present findings without interpretive statements.

4. Discussion

Comment 4.1: Emphasize novelty and clinical significance

Response: We expanded the Discussion to highlight the study’s novelty:

“This study pioneers a novel molecular subtyping framework for DKD by integrating glycosylation-related genes through bioinformatics and machine learning approaches[59]. Unsupervised clustering identified two distinct DKD subtypes that resolve disease heterogeneity, enabling personalized therapies such as antifibrotic agents for ECM-dominant patients. The identified hub genes demonstrated enhanced diagnostic performance (AUC >0.7) compared to conventional biomarkers. Mechanistically, we revealed glycosylation dysregulation drives macrophage/neutrophil activation, establishing a "glycosylation-immune crosstalk" axis. These findings reframe DKD pathogenesis as a glycosylation-mediated imbalance between extracellular matrix dynamics and immune regulation, providing precise therapeutic targets for precision medicine.”

Comment 4.2: Comparison with existing literature

Response: We incorporated the suggested reference (Fu et al., 2022) and contrasted our findings: While Fu et al. (2022) identified immune-related biomarkers in DKD using similar methods, our study uniquely links glycosylation-related genes to extracellular matrix dysregulation, offering complementary insights into DKD pathogenesis.

Reviewer #3

1. Abbreviations

Comment: Spell out all abbreviations at first mention. Response: All abbreviations have been defined at first use.

2. Abstract

Comment: Remove the repeated full stop in the Results section. Response: The duplicate punctuation in the Results section has been corrected.

3. Introduction

Comment: Clarify sentences in lines 36–42, 46, and 47–50 (now lines 44–54 in the revised manuscript).

Response: Sentences have been restructured for clarity:

Original (lines 44–46): "And O-linked glycosylation-SP1 modulating ENTPD5 expression through a negative feedback mechanism[8]"

Revised: "ENTPD5-regulated N-glycosylation of proteins in the ER to promote cell proliferation in the early stage of DKD, and continuous hyperglycemia activated the hexosamine biosynthesis pathway (HBP) to increase the level of UDP-GlcNAc, which driving a feedback mechanism that inhibited transcription factor SP1 activity to downregulate ENTPD5 expression in the late stage of DKD."

Comment: Replace citations to reviews (references 15–16) with primary sources for claims about glycosylation, inflammation, and oxidative stress.

Response: Primary studies have been added to citations:

Original: "Protein glycosylation maintains... by mediating inflammatory response and oxidative stress[15][16]."

Revised: [17] Radovani B, Gudelj I. N-Glycosylation and Inflammation; the Not-So-Sweet Relation. Front Immunol. 2022 Jun 27;13:893365. doi: 10.3389/fimmu.2022.893365. PMID: 35833138; PMCID: PMC9272703.

4. Materials and Methods

Comment: Report sources for kits/antibodies and software versions.

Response: Details added:

RNA extraction kit: FastPure Complex Tissue/Cell Total RNA Isolation Kit (Vazyme, cat# RC113-01).qPCR master mix: ChamQ Universal SYBR qPCR Master Mix (Vazyme, cat# Q711-02).

Primary antibody: CD45 (Abcam, cat# ab10558, 1:200 dilution).Software versions: R 4.2.3, limma 3.54.2, glmnet 4.1.7.

Comment: Rewrite Methods 1.8 (Immunohistochemistry) and 1.9 (Enrichment analysis) in reported form.

Response: Passive voice revised to active voice:

Original (Immunohistochemistry): "Fix kidney tissues in 4% paraformaldehyde..."

Revised: "Kidney tissues were fixed in 4% paraformaldehyde..."

5. Results

Comment: Include a workflow figure. Response: A workflow diagram has been added to the Supplementary Material, summarizing data acquisition, clustering, machine learning, and validation steps.

Comment: Add data points, label "Con" as "Control," and indicate statistical significance in Figure 8. Include ADAMTS1 and S100A12.

Response:

Revised:

"Con" replaced with "Control."

Significance markers added (ns: p>0.05; *: p<0.05; **: p<0.01; ***: p<0.005).

ADAMTS1 and S100A12 results are included (no significant differences noted).

6. Discussion

Comment: Address discrepancies in S100A12 and ADAMTS1 expression compared to prior studies. Propose further studies on EXT1.

Response: Added to Discussion:

"This inconsistency may arise from limitations in our animal model, including the small sample size (n=3 per group), which reduces statistical power to detect subtle changes, and species-specific regulatory mechanisms inherent to murine models. Additionally, disease stage heterogeneity (e.g., early vs. advanced DKD) and transient expression dynamics during pathological progression could mask gene activity."

"EXT1, a key enzyme in heparan sulfate biosynthesis, demonstrated robust diagnostic potential (AUC = 0.92). Future studies should explore its role in extracellular matrix remodeling and therapeutic targeting."

7. Primer List

Comment: Provide primer sequences.

Response: A primer table has been added to the Methods (Section 1.7)

We thank the reviewers for their valuable input. Please let us know if any additional revisions are needed. We are happy to further improve the manuscript.

Sincerely,

Ziyang Liu, Zengyuan Qin, Wenxin Bai, Shasha Wang, Chunling Huang, Na Li, Lei Yan, Yue Gu, Fengmin Shao

References

1. *Fu S, Cheng Y, Wang X, Huang J, Su S, Wu H, Yu J, Xu Z. Identification of diagnostic gene biomarkers and immune infiltration in patients with diabetic kidney disease using machine learning strategies and bioinformatic analysis. Front Med (Lausanne). 2022 Sep 29;9:918657. doi: 10.3389/fmed.2022.918657. PMID: 36250071; PMCID: PMC9556813.*
2. *Chen H, Su X, Li Y, Dang C, Luo Z. Identification of metabolic reprogramming-related genes as potential diagnostic biomarkers for diabetic nephropathy based on bioinformatics. Diabetol Metab Syndr. 2024 Nov 28;16(1):287. doi: 10.1186/s13098-024-01531-5. PMID: 39609849; PMCID: PMC11603941.*
