# Supplementary material for: Integrating bioinformatics and machine learning to elucidate the role of protein glycosylation-related genes in the pathogenesis of diabetic kidney disease
Source: PLoS One. 2025 Aug 18;20(8):e0329640. doi: 10.1371/journal.pone.0329640 (PMC12360563; doi:10.1371/journal.pone.0329640)
Supplement: Attachment — Submitted filename: Response to Reviewers2.docx [file pone.0329640.s004.docx]

Dear Dr. Ayipo and Reviewers,

Thank you for your careful consideration of our manuscript and the constructive feedback. We appreciate the opportunity to address the remaining concern raised in the second round of review.

Response to Reviewer #2. Comment: The authors stated that 10-fold cross-validation was added to the Methods section, but this was not found in the quoted Section 1.5.

We apologize for the oversight in the previous revision. The Methods section has been explicitly updated to describe the application of 10-fold cross-validation in all machine learning models. The relevant modification is as follows:

Revised in Section 1.5 "Identification of Hub Genes by Machine Learning":

"LASSO regression analysis was performed on gly-DEGs using the R package 'glmnet' to screen out genes with significant features. To minimize overfitting and enhance model generalizability, all machine learning models (LASSO, random forest, and SVM) were trained using 10-fold cross-validation, where the dataset was repeatedly partitioned into 10 subsets for iterative training and validation. Then, the 'randomForest' package was used to build a random forest model... " (Lines XX-XX, revised manuscript with track changes).

This addition clarifies the rigorous validation process applied to ensure the robustness of our machine learning results.

Response to Other Reviewers. Reviewer #1 and Reviewer #3 indicated that all previous comments were adequately addressed, and no further revisions were required for their concerns. We have double-checked the manuscript to ensure consistency with their suggestions.

Data Availability and Technical Accuracy. The data availability statement and statistical analyses remain consistent with PLOS ONE’s requirements, and all modifications are tracked in the revised manuscript.

Thank you again for your guidance in improving our work. We believe these revisions enhance the rigor and transparency of our study. Please let us know if any further adjustments are needed.

Sincerely,
Ziyang Liu et al.
